# Barriers and facilitators to physical activity among older Chinese adults in the UK: a qualitative descriptive study

**DOI:** 10.1186/s12877-026-07660-y

**Published:** 2026-06-04

**Authors:** Yang Yang, Nan Zhang, Lisa McGarrigle, Kimberly Lazo Green, Chris Todd

**Affiliations:** 1 School of Health Sciences, Faculty of Biology, Medicine and Health, The University of Manchester, Manchester, M13 9PL UK; 2 Manchester Academic Health Science Centre, Manchester, M13 9NQ UK; 3 National Institute for Health and Care Research Applied Research Collaboration - Greater Manchester (NIHR ARC-GM), The University of Manchester, Manchester, M13 9NQ UK; 4 Social Statistics, Global Network for Ageing Research On China/Chinese (GNARC), School of Social Sciences, Cathie Marsh Institute (CMI), Manchester China Institute (MCI), The University of Manchester, Manchester, M13 9PL UK; 5 Faculty of Science and Health, Atlantic Technological University, Co. Mayo, F23 X853 Ireland; 6 Policy Research Unit in Healthy Ageing, School of Health Sciences, Faculty of Biology, Medicine and Health, National Institute for Health and Care Research (NIHR), The University of Manchester, Manchester, M13 9PL UK; 7 Manchester University NHS Foundation Trust, Manchester, M13 9WL UK

**Keywords:** Physical activity behaviour, Barrier, Facilitator, Older Chinese adults, United Kingdom, Qualitative descriptive study

## Abstract

**Background:**

The Chinese community is one of the important ethnic groups in the UK, with a significant proportion entering older age. Older Chinese adults face ageing-related challenges and cultural barriers, which contribute to physical inactivity despite its well-recognised health benefits.

**Aims:**

To explore the barriers and facilitators to physical activity (PA) participation among older Chinese adults in the (United Kingdom) UK.

**Methods:**

We conducted 16 semi-structured interviews with 27 participants, using audio recordings collected either face-to-face or virtually. Interviews were conducted in one-to-one or paired formats, guided by a general, open-ended topic guide. Convenience sampling was used to recruit participants who self-identified as Chinese, were aged over 60, and resided in the UK. Data were analysed using thematic analysis with NVivo.

**Results:**

Participants’ PA behaviours fell into three categories: those who met (World Health Organization) WHO guidelines, those who were partially active, and those who were inactive. Factors influencing PA were mapped onto the COM-B (capability, opportunity, motivation-behaviour) mode. Identified barriers included poor health (e.g., lack of energy, pain, chronic conditions), limited PA-related knowledge, skills and self-Efficacy (e.g., low awareness of PA guidelines, insufficient practical know-how), restricted social opportunities (e.g., absence of exercise partners, limited social support, language and cultural barriers, time constraints), and limited physical opportunities (e.g., inaccessible environments, lack of appropriate programmes, limited access to PA information). Concerns about the potential risks or dangers of PA also deterred participation. Facilitators included strong beliefs in the benefits of PA—such as improved health, family harmony, social connection, and reduced loneliness—as well as automatic motivation through established habits, positive emotional experiences, and reinforcement.

**Conclusions:**

Older Chinese adults in the UK exhibit diverse PA patterns and face a range of barriers, underscoring the need to consider their varied needs and experiences when designing interventions. Factors influencing PA engagement align with the COM-B framework, highlighting the importance of addressing capability, opportunity, and motivation. These findings provide a practical evidence base to inform behaviour change interventions, supporting the development of culturally tailored strategies using the Behaviour Change Wheel to promote PA in this population.

**Supplementary Information:**

The online version contains supplementary material available at 10.1186/s12877-026-07660-y.

## Background

Physical activity (PA) is essential for health and well-being at all ages, with increasing benefits in later life. In older adults, regular PA helps prevent or manage age-related conditions, supports functional ability, and reduces the risk of falls and injuries [1, 2]. PA also improves cognitive function and reduces the risk of dementia [3]. Additionally, it promotes social interaction, helping to reduce loneliness [4]. Even low levels of moderate-to-vigorous PA are linked to a 22% reduction in mortality among older adults [5]. According to World Health Organization (WHO) guidelines [6], adults aged 65 and over should engage in at least 150 min of moderate or 75 min of vigorous PA per week, along with balance and strength exercises at least twice weekly, such as dancing or Tai Chi [6].

Although the benefits of PA are well-documented, physical inactivity is common worldwide. The prevalence of physical inactivity increases with age [7]. In the UK the most recent data from the Active Lives Survey conducted by Sport England 2021–2022 shows that PA levels generally decrease with age, with the sharpest decrease coming at age 75 +; around 41% of adults aged 75 and over were classified as being physically inactive [8]. The 2021 Health Survey for England also highlights the lower PA levels among older adults: for example, over one third (36%) of adults aged 65 and over were classified as being physically inactive [9]. 

Physical inactivity is more prevalent among older ethnic minority groups. For example, in the UK, among individuals aged 55–74, the Chinese ethnic group has a lower proportion of people classified as 'physically active' compared to the national average [10]. Only 58% of Chinese adults met the recommended guidelines, compared with 65% of White British and 71% of those of Mixed ethnicity [11]. The ethnic disparities in PA levels can be influenced by cultural factors, access to resources, and socioeconomic factors [12–14]. In the year ending June 2023, Chinese nationals were among the top three non-EU immigrant groups in the UK [15]. They are likely to encounter barriers to accessing health-related initiatives, including cultural and language barriers [16]. Older adults from the Chinese diaspora community typically maintain a strong affinity with Chinese culture, which influences their health beliefs and behaviours [17]. However, the limited scope of research restricts the comprehensive understanding necessary to devise targeted interventions specifically tailored to meet the needs of these diverse communities. Our recent systematic review aimed to identify the barriers and facilitators influencing PA engagement among older adults from the Chinese diaspora [18]. There was only one study targeting older Chinese adults in the UK, published in 2015 [16]. The study found that older Chinese adults often self-managed exercise based on cultural beliefs rather than professional guidance, which may pose risks without person-centred advice,however, insights into social and physical environmental influences on PA were limited.

Thus, it is imperative to conduct comprehensive, updated research that delves deeper into the lived experiences and perceptions of older Chinese adults concerning PA engagement within the UK context. This proposed research aims to bridge the knowledge gap by exploring the multifaceted aspects of participating in PA among this specific demographic. By uncovering the barriers and facilitators encountered by older Chinese adults, this study seeks to provide evidence-based insights that will inform the development of acceptable and effective interventions tailored to their unique needs and preferences.

### Research question

- What are the PA behaviours of older Chinese adults in the UK?
- What barriers and facilitators influence their participation in PA?
- What strategies might help them engage in PA?

## Method

A qualitative descriptive study design was used to ensure the findings reflect participants’ authentic experiences rather than researchers’ interpretations [19]. This approach is well-suited to exploring older Chinese adults' perceived barriers and facilitators to PA in the UK and identifying the features of culturally-specific PA promotion strategies. This study was reviewed and approved by the University of Manchester Research Ethics Committee (Ref: 2023–16653-28,843). This study is reported following the Consolidated Criteria for Reporting Qualitative Research (COREQ) checklist (Appendix 1).

### Participants

Participants were recruited from community settings in Manchester, UK, which has a substantial Chinese population [20]. Eligible participants were individuals aged 60 years or above who self-identified as being of Chinese ethnicity, spoke Chinese (Cantonese or Mandarin) and/or English, and had migrated to the UK from outside the country. There was no minimum duration of stay required, but participants had to be settled residents rather than visitors. Older Chinese adults who were in the UK for travel purposes (e.g. tourism or visiting family) were excluded, as well as those who were unable to provide consent.

### Recruitment

To ensure effective recruitment, a multi-faceted approach was employed, incorporating insights from previous studies [21] and recommendations from the Public and Community Involvement and Engagement (PCIE) advisors. We engaged with gatekeepers of Chinese community societies and Chinese Christian churches, to facilitate direct contact with potential participants. Advertisements were posted in locations frequented by older Chinese adults, such as Chinese community centers, churches, and supermarkets. Social media outreach, particularly through WeChat, was also utilised to maximise visibility within the Chinese community. In addition, participants were encouraged to refer others who might be eligible and interested in the study.

All recruitment materials, whether printed or digital, were prepared in both traditional and simplified Chinese, as well as English. Potentially eligible participants were invited to take part in an assessment of their eligibility after providing consent. Written consent was obtained for face-to-face interviews. For online interviews (Teams or Zoom), participants either typed their name on the consent form and confirmed consent by email or provided verbal consent after the form was read aloud, which was recorded and stored separately.

### Sampling and sample size

Convenience sampling was used to identify the initial study subjects, and eligible participants who volunteered to participate were included. Efforts were made to recruit participants from different age groups, genders, languages, and activity levels to maximise variation.

Based on previous similar studies [16] and the literature [22, 23], this study aimed to recruit approximately 15–30 older Chinese adults. Data saturation [24] was defined as the point at which no new data emerged regarding perceived barriers and facilitators to PA participation or recommendations for ideal PA promotion interventions.

### Data collection

Demographic and health-related information was collected using a pre-defined questionnaire, which participants completed prior to each interview (Appendix 2). An audio-recorded semi-structured interview method was employed to collect qualitative data [25]. The interviews were conducted by the lead author (YY), who is of Chinese heritage, fluent in Mandarin, and assisted by a research assistant (LSH), fluent in Cantonese and Mandarin, both familiar with Chinese culture and trained in qualitative research methods. Participants were given the option to attend a one-on-one interview or participate with their family and friends. The interviews lasted between 30 and 90 min. The initial topic guide was presented in Appendix 3. The interview topic guide remained flexible and was regularly updated throughout the data collection process as new themes or areas of interest emerged during the interviews. Data collection and analysis were performed concurrently, with ongoing discussions among the research team to review the findings.

### Data analysis

NVivo 12 software was used to conduct data analysis following the thematic analysis method. The audio recordings of the interviews were transcribed by the lead author (YY for Mandarin) or research assistant (LSH for Cantonese). The interviews were analysed in their original language rather than translated into English to preserve the authenticity and nuance of participants' expressions, ensuring that cultural and contextual meanings were accurately captured and interpreted. Themes were labelled in English, and quotations were translated into English only at the reporting stage. Data analysis was conducted by the lead researcher (YY), with ongoing discussions of the emerging themes with supervisors to ensure rigor and credibility.

Thematic analysis was used to explore participants’ experiences related to PA engagement [26]. The analysis began inductively, allowing themes to emerge from the data, and later transitioned to a deductive approach by mapping findings onto the COM-B model of behaviour [27], which includes capability, opportunity, and motivation. The analysis followed Braun and Clarke’s [26] six-phase framework: familiarisation, coding, theme development, theme review, theme definition, and final reporting. Conceptual models were developed based on Naeem et al. [28] to visually represent the thematic analysis process.

### Reflexivity

In this study, we employed several strategies to promote reflexivity as suggested by Olmos-Vega et al. [29]. Firstly, the lead researcher maintained a research journal to document her perspectives and decision-making process at each stage of the study. A positionality statement was prepared to reflect on how her personal experiences had impacted the research process and potentially shaped the results. Furthermore, we incorporated member reflections as an additional measure to ensure the rigour of this qualitative study.

### Positionality statement

The lead researcher, a Chinese PhD student with a nursing background and experience in healthy ageing research, shares cultural and linguistic commonalities with participants and has personal insight into immigrant life in the UK. The research assistant (LSH) is also a Chinese PhD student with a nursing background and experience working with older Chinese adults in the UK. These shared characteristics facilitated access, communication, and sensitivity to participants’ perspectives, although they may also have increased the risk of overlooking routine issues, and age differences may have shaped interpretations of older participants’ experiences. While an open-ended interview approach was used to minimise bias, data interpretation may still have been influenced by the lead researcher’s prior academic work. The supervisors (NZ, KG, LM, and CT) bring diverse disciplinary perspectives and extensive experience in healthy ageing research and provided oversight throughout the study.

## Results

### The participants

A total of 27 participants were interviewed across 16 sessions: ten were one-on-one interviews, while six involved two or three participants interviewed together. The majority of these interviews (12 out of 16) were conducted face-to-face, with only 4 conducted online. All interviews were conducted in the participants’ first language (Mandarin or Cantonese). Interviews took place from July 2023 to March 2024.

Participant characteristics are presented in Table 1. The 27 participants (mean age = 70), were mostly female (78%), married (78%), and retired (89%), and had on average lived in the UK for 31.6 years (range: 1–50). The primary languages spoken were Cantonese (59%) and Mandarin (41%). In terms of self-reported health, 63% rated their health as good. PA levels varied: 26% exercised less than once per week, 30% two to three times per week, and 44% more than four times per week.

**Table 1:** Characteristics of the study sample (*n* = 27)

Characteristics	*n* = 27
Mean age in years (range)	70 (60–97)
Mean years living in the UK (range)	31.6 (1–50)
Gender, n (%)	
Female	21 (78%)
Male	6 (22%)
Marital status, n (%)	
Married	21 (78%)
Divorced	1 (4%)
Widowed	5 (18.5%)
First language, n (%)	
Mandarin	11 (41%)
Cantonese	16 (59%)
Occupation, n (%)	
Retired	24 (89%)
Part-time job	2 (7%)
Full-time job	1 (4%)
Self-report health, n (%)	
Very Good	6 (22%)
Good	17 (63%)
Poor	4 (15%)
PA level, n (%)	
< 1 time each week	7 (26%)
2–3 times each week	8 (30%)
> 4 times each week	12 (44%)

## Interview findings

### The PA behaviour among older Chinese adults in the UK

The PA behaviours of the participants were categorised into three PA groups in relation to WHO guidelines was derived from qualitative interview data. These groupings were based on participants’ self-reported PA behaviours, including the type, frequency, and intensity of activities discussed during the interviews:

- Individuals meeting WHO PA guidelines for older adults, engaging in aerobic activities (e.g., brisk walking, swimming), and recommended balance and strength exercises.
- Individuals self-report being physically active but not meeting full guidelines, focusing on aerobic activities (e.g., walking) but lacking balance or strength training.
- Individuals who are physically inactive and primarily sedentary.

The types of PA behaviours mentioned by the participants included walking, Tai Chi, dancing, swimming, gardening, housework, strength training, table tennis, tennis, cycling, and stationary bike. Among these, the most commonly reported activities include walking, Tai Chi, and dancing. Walking was noted as the most convenient activity, Tai Chi as a traditional Chinese exercise, and dancing as an enjoyable group activity. Access to group exercise encouraged engagement in shared activities such as dancing and Tai Chi, whereas geographic distance often led individuals to favour more solitary activities, including walking and swimming.

### The factors that influence PA of older Chinese adults in the UK

**Fig. 1:**
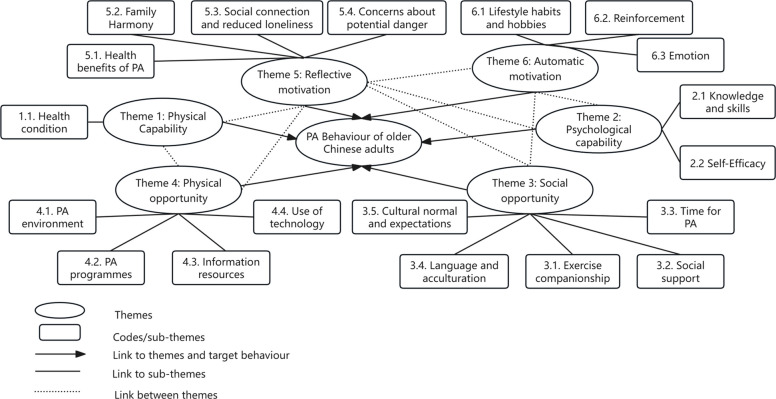
Thematic map demonstrating six themes related to the PA behaviour of older Chinese adults

The factors influencing PA among older Chinese adults in the UK were categorised into six themes, corresponding to the six sub-dimensions of the COM-B model (Fig. 1). Thematic approaches for capability (Themes 1–2), opportunity (Themes 3–4), and motivation (Themes 5–6) are presented in Appendix 4, 6, and 7, respectively.

#### Theme 1: physical capability- health conditions and functional limitations

Health conditions are reported as the main barrier by some participants, especially those of a very advanced age. They reported a lack of energy for PA participation, while pain and chronic conditions like arthritis often limit their ability to engage in PA, particularly in exercises requiring substantial exertion. As one participant expressed it*: "But now I’m just too old to exercise; I simply don’t have the strength for it anymore."* (Interview 16, P-27, Female, 87 years old). Similarly, another participant highlighted illness-related barriers “*When I was younger, I enjoyed playing table tennis, but as I got older my health declined. After I had a stroke, I could no longer walk, let alone exercise. Health problems become a major barrier as you age*” (Interview 15, P 25, 86 years old). Health problems and advanced age can also reduce motivation for PA participation*. "At this age, and with my poor health, nothing feels meaningful anymore. I know PA is good for health, but I just don’t care anymore. I just want to be comfortable and spend most of my time in bed."* (Interview 16, P-27, Female, 87 years old).

Health conditions were less commonly reported among younger and healthier participants; however, some still noted that emerging health issues had prevented them from exercising. “*I like* dancing*, but now I have a problem, you know, sometimes I feel dizzy. It has been 2 months, so I haven’t come to dance since then*” (Interview 5, P-8, Female, 67 years old). Younger older adults also acknowledged that health would become a major obstacle as they age. *"We are still younger, under 70, so we need to practise now. However, when we get older, health will be the biggest problem, and no one can help." *(Interview 4, P-4, Female, 67 years old)*.*

#### Theme 2: psychological capability: PA knowledge, skills, and self-efficacy

PA knowledge, skills, and self-efficacy shape psychological capability for PA

- Theme 2.1 PA knowledge and skills

Many participants reported a lack of knowledge and skills needed to meet PA guidelines, including the recommended amount and types of exercise for older adults. Many assumed that walking alone was sufficient. As one participant stated, “*I don’t know a lot about exercise… I think I am active—I walk every day, and I believe that’s enough….*” (Interview 1, P-1, Female, 60 years).

Due to this lack of knowledge, participants often avoid higher-intensity activities, which may lead to them not meeting the recommended PA levels required to achieve optimum health benefits. “Older adults should not walk too fast. Many seniors have heart problems or high blood pressure, and too much activity can make the heartbeat too fast, which is not good” (Interview 11, P-20, Female, 60 years). Additionally, some participants reported lacking the skills to perform the exercises. As one participant noted, “I just go for walks; I don’t know how to do other exercises. At our age, learning new sports is quite difficult. "Table tennis is fine—many older Chinese adults enjoy it, but I don’t know how to play.” (Interview 6, P-11, Female, 68 years).

- Theme 2.2 self-efficacy

Self-efficacy was closely shaped by perceived physical capability, which reduced confidence in engaging in PA independently. Low self-efficacy may reduce willingness to engage in PA, as individuals with poorer health often require accessible facilities (e.g., nearby toilets) and may be more likely to remain homebound. As one participant noted: *“There must be a toilet available. Many older adults, especially women, experience urinary incontinence, which discourages them from leaving home*” (Interview 12, P-21, Female, 80 years). Another participant shared similar concerns, *“I’m too old to go out alone. I only go out for lunch or a walk when my daughter visits”* (Interview 16, P-27, Female, 87 years).

To enhance psychological capability, participants suggested strategies aimed at improving their knowledge and confidence: “*I think you need to tell them the importance of exercise. They also need to know what kind of exercise to do, how much, and how to do it. Otherwise, many won’t exercise at all*” (Interview 8, P-15, Female, 62 years).

#### Theme 3: social opportunity: companionship, social support, time, language and culture

Common social opportunity factors included exercise companions, language and cultural influences, social support, and time availability.

- Theme 3.1 exercise buddy/group

Experiences of exercising with companions varied depending on participants’ access to, and proximity to, Chinese community organisations and culturally similar exercise partners. Many participants preferred exercising with a companion, but older Chinese adults in rural UK areas often struggle to find culturally and linguistically similar partners, leading to isolation and reduced PA participation. As one participant shared: *“For me, the biggest challenge is finding exercise buddies. There aren’t many older Chinese people around, and it’s more interesting to have company.”* (Interview 1, P-1 Female, 66 years old). Another echoed this concern, highlighting safety issues when exercising alone: *“The riverside is secluded, and walking alone feels unsafe. As seniors, we feel more secure with a companion—someone who can help if needed. I don’t dare go alone.”* (Interview 13, P-12, Female, 62 years old).

Some had even considered forming exercise groups but found it impractical. *“I’ve thought about organising a group for seniors to exercise together, but there aren’t many Chinese people around here.”* (Interview 2, P-2, Female, 66 years old). Others lost interest in PA due to the lack of a shared practice environment. *“I used to practice Tai Chi…. But in the UK, practicing alone feels meaningless. It’s not like in China, where we could train together and learn from each other, so I lost interest.”* (Interview 3, P-3, Male, 60 years old). On the other hand, being part of an exercise group can facilitate PA participation. “*Because we have the Xinhua Association, there are all kinds of clubs, like badminton club and dancing club. I don’t feel there are any major barriers…*” (Interview 8, P-15, Female, 62 years old).

Although most participants preferred exercising with others, some enjoyed solo workouts. One participant shared: “*I prefer exercising alone at home. I follow instructional videos—no interruptions, just the comfort of my own space. I can pause whenever I want*” (Interview 12, P-21, Female, 80 years old). Others found group exercise challenging due to pace differences. “*Even walking with friends doesn’t always work for me. I walk slower and often have to jog to keep up. It’s exhausting… That’s why I prefer walking alone at my own pace*” (Interview 11 P-20, Female, 60 years old).

- Theme 3.2 support for PA

Participants reported that support from family, community, or healthcare professionals facilitated PA engagement.

Family support appears to be an important factor in motivating older adults to stay active. One participant shared: *"My grandson used to call every morning to remind me to exercise, so I’d walk around my room. But he hasn’t called lately, maybe he's busy, so I’ve stopped exercising."* (Interview 16, P-27, female, 87 years old). Similarly, spousal encouragement can influence PA participation. Another participant mentioned: *"If my wife reminds me, I’ll exercise—but without her, I just sit and watch TV all day. I don’t have much energy for it."* (Interview 15, P-25, Male, 86 years old). Beyond verbal encouragement, practical support, such as transportation, also facilitates participation. One participant shared: “*My friend and I have danced together for years. Her husband was initially against it, but seeing her happiness, he became supportive—now he even drives her and waits. It’s also improved their family relationship."* (Interview 7, P-14, Female, 72 years old).

Community resources and healthcare professionals also play a role in promoting PA. Some participants valuing organised exercise programmes in their living environments. *"I live in a senior apartment where weekly group exercises are organised. It's kind of them, and with professionals leading, it feels like a great opportunity for older adults."* (Interview 12, P-21, Female, 80 years old). Similarly, responsive community management enhances PA opportunities by addressing residents' needs. One participant noted: *"The community manager is very supportive. We requested table tennis tables—first there were none, then two, then three, and now four. That’s real support!"* (Interview 9, P-17, Male, 70 years old). Healthcare professionals also advocate for PA, particularly for older adults. *"The family doctor recommends doing some manageable exercise—you can’t just do nothing, even simple movements help."* (Interview 16, P-27 Female, 87 years old).

- Theme 3.3 time for PA

Participants generally report having more time for exercise after retirement. “*Now that I’m retired, I have plenty of time to exercise—it all comes down to whether I want to or not*”. (Interview 1, P-1, Female, 60 years old). However, some still experience a lack of time due to ongoing work and family obligations, which limit their ability to engage in PA. For those who are still working, long hours and demanding jobs make it difficult to prioritise exercise. One participant shared: *"When you’re working—like me, six days a week—there’s no time for exercise. I’m always busy cleaning at my employer’s house, so how can I go out and exercise?"* (Interview 11 P-20, Female, 60 years old). Similarly, caregiving responsibilities can take up considerable time, especially for grandparents who look after their grandchildren. As one participant explained: *" I enjoy hiking or short-distance trips with friends, but I can’t—I'm busy caring for my grandchildren and only have free time while they’re at school. so these options are not really feasible for me."* (Interview 13, P-12, Female, 62 years old). Additionally, some participants prioritise supporting their adult children’s businesses over their own PA. One participant described their situation: *"I haven’t exercised because I don’t have time. My children run a small business and are very busy, so I help them—there’s no time left for exercise."* (Interview 14, P-23, Female, 68 years old).

- Theme 3.4 language and acculturation experiences

Language influences PA behaviour through different acculturation pathways. For some participants, limited English reflected low acculturation to the host society, restricting access to mainstream PA information and opportunities. One participant shared, *“I never look at the NHS website because I cannot read English.”* (Interview 1, P-1, Female, 60). Another noted, “*Some communities share leaflets about local activities, but they’re in English, so some older adults may not understand them. Still, it’s a good idea*.” (Interview 2, P-2, Female, 66). As one older participant summed up, “*Our biggest disadvantage is the language barrier. If I spoke English, I could join any exercise activities in the UK.*” (Interview 15, P-25, Male, 86). Additionally, some participants preferred activities requiring minimal communication. As one noted, “*I like swimming because you don’t need to talk—just a smile is enough*.” (Interview 5, P-7, Female, 74).

However, for others, strong attachment to the heritage culture reduced the perceived need to engage linguistically with the wider community. One participant explained, *“I don’t see language as a big issue. I have many Chinese friends here, and we always play [dancing] together, so I don’t really need to speak English.”* (Interview 6, P-13, Female, 63). This sentiment was common among older Chinese individuals who, despite living in the UK for decades without speaking English, remain content within Chinatown, maintaining familiar lifestyles. Language and cultural barriers also existed within the Chinese community, particularly between Mandarin- and Cantonese-speaking groups: “*Early Chinese immigrants have many activities, but they speak Cantonese, and I speak only Mandarin. I just don’t feel I fit in. Exercise should be enjoyable, but if you feel out of place, you won’t want to join.*” (Interview 15, P-25, Male, 86).

- Theme 3.5 cultural normal and expectations

Beyond language, many participants found cultural differences to be an even greater challenge. As one participant explained, *"Yes, it’s different. It feels more comfortable when talking with Chinese friends. I think my English is okay, but I still prefer exercising with my Chinese friends—it just feels closer and more connected. It’s just in my roots—I prefer socialising with Chinese people."* (Interview 5, P-9, Female, 65 years old). These cultural differences also shape exercise preferences. As another participant described, *"Chinese people enjoy chatting about daily life, while British people tend to value privacy. They often go to the gym, but we prefer group activities like dancing or Tai Chi—it’s not just exercise, but also a chance to socialise."* (Interview 5, P-7, Female, 74 years old).

In addition, Chinese cultural values, including modesty, age-appropriate behaviour, and social perception, can also influence PA participation. Some older adults expressed hesitation or discomfort toward certain activities they considered inappropriate for their age. As one participant noted: *“Many Older Chinese adults feel embarrassed, thinking, ‘At this age, dancing?’ So they just go for a walk or do housework and consider that their exercise”* (Interview 7, P-14, Female, 72 years old). Another said *“I’m not conservative—I’m quite outgoing. But I’m not into square dancing. As you get older, you should be more low-key. I just don’t feel comfortable doing it.”* (Interview 11 P-20, Female, 60 years). Furthermore, cultural expectations within family structures also contribute to PA-related embarrassment. One participant shared how they once enjoyed dancing but eventually stopped due to feeling self-conscious around family members: *“Sometimes I play music and dance at home, but I feel embarrassed when my son-in-law is around.”* (Interview 14, P-23, Female, 68 years old).

#### Theme 4: physical opportunity: environmental, programme, and resource access

The PA environment and available programmes were the most frequently mentioned physical opportunity factors, alongside information resources and technology.

- Theme 4.1 PA environment

Outdoor environment, such as proximity to parks, community safety, and traffic conditions, plays a crucial role in encouraging walking and attending other activities. Many participants reported a lack of suitable exercise spaces: *"There is nowhere to do exercise*. *The parks have playgrounds* but no *fitness equipment, it's all grass. When it rains, it gets very muddy. On rainy days, the parks are unusable because of the mud."* (Interview 13, P-12, Female, 62 years old). Beyond accessibility, safety concerns further discourage outdoor exercise, particularly in the evenings. An elder participant shared:* "An elderly lady was robbed before—she was wearing earrings and other jewellery. So I only go out for a walk during the day. The security isn’t great—it’s not as safe as in China…"* (Interview 12, P-21, female, 80 years old).

The availability of indoor space also shapes participants’ PA behaviour. Many expressed the need for accessible and affordable venues for group exercise. One participant explained*, "The main issue is there’s no place for us. If there were an indoor space, we could dance or exercise together. Maybe the church has a space, but I don’t know—so I just walk in the park."* (Interview 13, P-12, Female, 62 years old)*.* One participant reflected on their experience*, "We used to dance in the park on the basketball court, and the community was supportive—they even removed a hoop for us. But with frequent rain, it wasn’t sustainable. Later, a Chinese language school let us use a storage room for £1–2 per session, but we’re unsure about its safety. What we really need is a proper space to gather."* (Interview 5, P-9 female, 65 years old).

The group exercises provided by Chinese associations were usually held in city centres, but many participants lived in suburban areas. As a result, they needed to travel to attend group PA, making transportation a major challenge, especially for those living further away. One participant noted the financial burden: *"Transport is expensive—£4 a day adds up. I have to pay for every trip, and free travel only starts at 67."* (Interview 13, P-12, Female, 62). Another pointed to time: *"Transport is a hassle. We live far apart, and it takes me over an hour to get here. Time is the main issue."* (Interview 5, P-9, Female, 65).

- Theme 4.2 PA programme

The availability of PA programmes is another factor influencing participation. "*Unlike in China, there are no activities like square dancing here, and exercise options are limited. I wish we had a Chinese community club to dance, socialise, and make friends—but there isn’t one.*" (Interview 11 P-20, Female, 60 years). Participants highlighted the need for exercises that are both accessible and appropriate for older adults, with one stating, *"For older adults, I think it’s really important to choose simpler and safer exercises."* (Interview 11 P-20, Female, 60 years). Professional guidance was also seen as crucial for maintaining trust and commitment: *"It’s best to have a professional to guide you; otherwise, people won’t fully trust you or stay committed to exercising with you."* (Interview 4, P-6, Female, 74 years). Enjoyment and affordability were key considerations as well. One participant explained, *"I don’t exercise much, but I’d join if it’s fun. Two things matter: it has to be free, and it has to be interesting. I wouldn’t go to the gym—even for free—it’s boring. I’d rather dance, sing, or go on trips with others."* (Interview 15, P-25, Male, 86 years old).

- Theme 4.3 Information resources

Information resources also influence participants’ PA participation. Especially for newly arrived participants, navigating PA opportunities in an unfamiliar environment was particularly challenging: “*I just moved to the UK, so I don’t know many people or where I can exercise. If I knew, I would be very willing to participate…*” (Interview 13, P-12, Female, 62 years old). Despite these barriers, some saw digital resources as helpful alternatives, particularly for those unable to attend in-person sessions: “*…you can learn fitness exercises and dance routines online. Look, I just place the phone here and follow along. If I find good ones, I save them and share them with my friends.*” (Interview 7, P-14, Female, 72 years old).

Instead of creating new activities, one participant suggested focusing on compiling and sharing information about existing PA opportunities: *"Maybe you don’t even need to set up new activities… just putting all the information together would be really helpful."* (Interview 8, P-15, Female, 62).

- Theme 4.4 using technology

Technology played a role in some participants’ PA behaviour, though attitudes varied. While some found digital tools helpful for exercise and motivation, others expressed concerns about difficulty, privacy, or effectiveness. For example, the popular social media platform WeChat was frequently mentioned. One participant said, "*I like peace and staying home. I follow short WeChat videos to exercise, but I’m not sure if they’re good or not*." (Interview 12, P-21, Female, 80). Another shared, "*WeChat has a step tracker where friends check each other’s progress. I set a 6,000-step goal in summer—it keeps me motivated.*" (Interview 8, P-15, Female, 62).

Online exercise sessions became more common during COVID-19, but not all participants found them appealing. One shared, "*We did online Tai Chi during the lockdown, but it wasn’t very interesting or easy to follow. In-person classes are more fun, and the teacher can correct mistakes*." (Interview 8, P-15, Female, 62). Others struggled with technology use: "*I have a smartphone but don’t know how to use it—I only contact family and rarely check it*." (Interview 16, P-27, Female, 87). Concerns about privacy also discouraged some from using digital tools: "*I had a smartwatch from the Chinese community to track steps, but when I heard it could leak personal info, I stopped using it.*" (Interview 15, P-25, Male, 86).

#### Theme 5: reflective motivation: beliefs, social interaction, and concerns

Reflective motivation was driven by beliefs in the benefits of PA for health, wellbeing, independence, and family roles, although some participants expressed concerns about potential negative effects.

- Theme 5.1 belief in the health benefits of PA

First, PA was seen as crucial for maintaining overall health and preventing illness. One participant emphasised this, saying, *"For us seniors, exercise is really important. If you don’t move and just sit around all day, your body gets weaker over time. But if you keep exercising, you feel more energetic and don’t get sick as often."* (Interview 10, P-19, Male, 71 years old). Some participants turned to exercise after experiencing health issues, realising the importance of staying active. *"When I first came to the UK, I stayed home all the time and felt awful—back pain, sciatica, sore shoulders. I realised I couldn’t just sit around, so I decided to start exercising."* (Interview 10, P-18, Male, 67 years). The traditional Chinese belief in the health benefits of movement was also reflected in participants' attitudes. As the old Chinese saying goes*, “Walk a hundred steps after a meal and live to ninety-nine.' If you want to stay healthy and live long, you have to keep exercising."* (Interview 1, P-1, Female, 60 years old). Participants also highlighted the mental health benefits of PA. As one participant explained: *"If you stay home all the time and don’t talk to anyone, your brain slows down. Getting out, moving around, and chatting with people helps keep your mind sharp—and may even prevent dementia. Stay in too much, and you just feel sluggish, haha!"* (Interview 5, P-8, Female, 67 years old).

Some participants held alternative beliefs, viewing longevity as determined more by genetics than PA. One said, "*It’s all destiny or genetics! My friend’s mum never exercises and she’s in her 90s.*" (Interview 2, P-2, Female, 66). Another questioned the value of movement: "*Some believe moving less leads to a longer life—too much activity wears you out. Look at turtles—they barely move but live long*." (Interview 11, P-20, Female, 60 years old).

- Theme 5.2 belief in the benefit of family harmony

Participants highlighted the role of PA in fostering family harmony through multiple pathways. Maintaining health and independence was a primary motivation, as it helped reduce the caregiving burden on family members: "*Exercise keeps you healthy. Otherwise, if you’re always sick, it’s hard on your family and kids. They’re already busy, and I don’t want to be a burden.*" (Interview 7, P-14, Female, 72). For some, staying active was essential to fulfilling caregiving responsibilities: "*I exercise to stay healthy. It’s just the two of us here, and I need to care for my husband after his stroke.*" (Interview 15, P-24, Female, 84).

PA was also seen as a way to ease children’s concerns and offer emotional reassurance: "*When they know I’m exercising, the kids feel more at ease. If I’m unwell, they worry, and it affects their work.*" (Interview 2, P-2, Female, 66). In addition, PA helped strengthen spousal relationships by facilitating shared time and communication: "*At home, my wife and I rarely talk, but when we go for a walk or hike, we talk a lot. It’s good for our relationship.*" (Interview 10, P-19, Male, 71).

Beyond the family unit, participants also emphasised the broader psychosocial benefits of PA, such as promoting social engagement and emotional well-being: *"When you’re healthy, you can join social activities and feel happy. If you’re always sick, it’s hard for both you and your family."* (Interview 7, P-14, Female, 72).

- Theme 5.3 PA is a way to meet people and reduce loneliness

Social interaction emerged as both a social and motivational factor influencing PA participation. For many participants, PA was not only a means to stay active but also an important opportunity for social engagement, particularly in the context of migration and ageing. It served as a natural platform for building connections, forming friendships, and reducing loneliness. As one participant explained: "*Exercise is one thing, but for me, it’s also about socialising. Joining a walking group is a great way to meet people and chat. Without social interaction, life feels lonely and kind of pointless.*" (Interview 6, P-11, Female, 68).

For some, the enjoyment of PA was closely tied to the social bonds formed through group activities. One participant described: "*I like ping pong. Our group gets along really well—sometimes we go out to eat after playing. It’s not just about the game, it’s about having fun with friends.*" (Interview 9, P-16, Male, 71).

- Theme 5.4 worry about the potential danger

In contrast, concerns about safety and a belief in the benefits of a quiet lifestyle acted as barriers to PA participation. Fear of injury, particularly fear of falling, was a common concern among older participants. One shared, "*I’m afraid something might go wrong if I try (exercise). If you fall, you might not be able to get back up. I’d rather just watch TV*." (Interview 15, P-25, Male, 86). Past experiences reinforced these fears for some. As one participant recounted: "*I usually stay home and watch TV. It’s not that I don’t want to exercise—I’m just too afraid. I live alone, and if I fell, no one would even know. My husband also fell, went to the hospital, and passed away soon after. So I’m really scared of falling.*" (Interview 16, P-27, Female, 87).

#### Theme 6: automatic motivation- habit formation and emotional responses

Regular PA participation may foster automatic motivation, characterised by habitual, emotion-driven responses shaped by positive experiences and reinforcement.

- Theme 6.1 lifestyle habits

Many participants described PA as an ingrained habit. One explained, "*My husband and I always go for a walk after dinner if the weather is good. It’s already a habit—if we don’t move, we feel uncomfortable. A little exercise helps with digestion.*" (Interview 1, P-1, Female, 60). For some, skipping a day of exercise created a sense of unease: "*I swim every day, and if I skip a day, I don’t feel right.*" (Interview 5, P-7, Female, 74). Beyond routine, some engaged in PA to pass the time, especially in later life when daily responsibilities had decreased. Walking was commonly mentioned: "*We’re all getting older and don’t have much to do, so going out for a walk kills time. It’s better than sitting at home watching TV. If the weather’s nice, I walk every day.*" (Interview 4, P-4, Female, 67).

Participants can also engage in PA due to intrinsic enjoyment and personal interest, demonstrating a natural desire to explore techniques and refine skills purely for the sense of fun and satisfaction it brings. For example, one participant explained: *“For me, it’s all about interest…. I enjoy the technical side of ping pong, watching videos and practising to refine my skills. It’s really fun.”* (Interview 10, P-18, Male, 67 years).

- Theme 6.2 reinforcement

Experiencing tangible health benefits reinforced participants’ motivation to engage in PA. Several described improvements in physical function that encouraged continued participation. One participant reflected, "*Before the pandemic, I hardly exercised. I couldn’t run for a bus. But after playing ping pong, I can now sprint 100 m to catch one, and it feels amazing!*" (Interview 10, P-18, Male, 67).

Others noted pain relief as a motivating factor. "*Exercise makes me feel better. I used to have leg pain, and now it’s much improved.*" (Interview 12, P-21, Female, 80). Swimming was highlighted for its therapeutic effect: "*Swimming is the best. I had shoulder pain, and just one session would ease it. Now, whenever I feel uncomfortable, I swim and feel much better.*" (Interview 13, P-12, Female, 62).

In addition to physical relief, participants linked PA with maintaining appearance and vitality. "*Exercise keeps you in shape and makes you look younger. People say I don’t look like I’m in my 70s! That’s because I keep moving. It’s not just about looks; it makes me feel good too.*" (Interview 7, P-14, Female, 72).

- Theme 6.3 emotion

PA was also seen as a way to manage emotions, offering joy and stress relief. Participants described how activities such as dancing and walking improved their mood. One noted, "*Dancing together lets us let out our emotions and forget our worries. With the music playing, it just feels so good! It makes you happy.*" (Interview 4, P-5, Female, 65). The social aspect amplified this effect: "*When you exercise with a group, the energy is different. Everyone’s excited and happy. You just want to join in again!*" (Interview 5, P-8, Female, 67). Spending time outdoors also supported emotional well-being. "*When you’re feeling down, a walk can change everything. Seeing the mountains, rivers, and flowers makes you feel better*." (Interview 4, P-5, Female, 65).

However, emotional distress could also reduce motivation for PA. Grief, particularly after the loss of a loved one, made it difficult for some to continue previous routines: "*I know exercise is important, but I just don’t have the mood. Since my partner passed away, nothing feels meaningful. I used to care about eating well and staying active, but now I don’t feel like doing anything.*" (Interview 14, P-23, Female, 68).

## Discussion

This study explored the barriers and facilitators to PA participation among older Chinese adults in the UK, aiming to inform effective PA promotion strategies. Interviews with 27 participants revealed a wide range of PA behaviours. Some participants were highly active and met the recommended guidelines, others engaged in light walking but did not reach the recommended levels, and some had adopted predominantly sedentary lifestyles. The factors influencing their PA participation were categorised into six themes, aligned with the Capability, Opportunity, and Motivation components of the COM-B model.

### Capability

Similar to other ageing populations, underlying health conditions influence the PA behaviours of older Chinese adults in the UK [30]. Chronic diseases and poor self-assessed health are associated with a higher prevalence of functional disability, reducing participants’ capability to engage in PA [31]. Additionally, physical health impacts self-efficacy and outcome expectations, both of which reduce motivation to exercise [32]. Furthermore, conditions such as pain and low energy limit opportunities for more demanding forms of exercise, restricting participation in higher-intensity physical activities [33]. In addition, culturally shaped health management, including health literacy and access to support, may also influence how health concerns affect PA engagement.

Similar to the general ageing population in the UK, another factor influencing the PA behaviour of older Chinese adults is skills and knowledge about PA [34]. Findings from the literature on the relationship between guideline knowledge and PA behaviour remains mixed. While one study found no association between PA guideline knowledge and higher PA levels in older adults [35], other research indicates that inadequate health literacy is marginally significantly associated with poor compliance with PA guidelines [36]. Consistent with previous research [37], many participants failed to meet PA guidelines due to limited awareness and the belief that walking alone is sufficient exercise. Participants were often unaware of the need for strength and balance training and lacked the skills to perform these activities. In addition, older Chinese adults’ PA behaviours were also shaped by traditional Chinese cultural beliefs, often leading to a preference for gentle activities and caution towards moderate or higher intensity PA.

### Opportunity

While language barriers have frequently been identified as obstacles for older adults from ethnic minority groups in accessing local PA opportunities [14, 18, 38], participants in this study expressed more nuanced views shaped by different acculturation pathways [39]. Many participants, including those who had lived in the UK for decades, remained closely connected to Chinatown communities, which actively facilitated their PA participation. This supports the concept of “aging in Chinatowns” described in earlier research [17], wherein such communities offer comprehensive support systems, ranging from practical and linguistic assistance to social connection, emotional comfort, and cultural continuity. These culturally embedded experiences appear to shape and sustain PA behaviours among older Chinese adults [17].

In Manchester, the research site, Chinatown and several long-established Chinese associations played a vital role by providing culturally tailored PA programmes for Chinese seniors. Participants engaged with these organisations reported fewer opportunity-related barriers and greater satisfaction with their PA routines. In contrast, those living in more dispersed areas or unaware of such associations faced challenges in accessing appropriate programmes or finding exercise companions from similar backgrounds.

Many Chinese associations in the UK primarily use Cantonese, reflecting earlier migration patterns. Mandarin-speaking participants often found it difficult to engage with these groups, highlighting a language divide within Chinese communities that has received limited attention in previous research.

In addition to social opportunities, physical opportunities also influenced participants’ PA behaviour, with access to suitable facilities and safe environments shaping participation [40]. However, for older Chinese adults in the UK, a particularly prominent barrier is the lack of culturally tailored exercise programmes and accessible venues, which consistent with findings among other ethnic groups in the UK [38]. Due to language and cultural barriers, older Chinese adults are less likely to use mainstream local community centres or PA programmes and instead tend to engage in activities organised by Chinese community organisations, which are limited in number and typically located in city centres. One PCIE participant (a manager of a Chinese association) also highlighted inequities in government financial support for Chinese communities, suggesting that limited funding further restricts access to PA opportunities.

Family may play a complex role in shaping participants' PA behaviour in Chinese communities in our study. Consistent with the general ageing population, family support, such as encouragement and practical assistance, serves as a key facilitator for PA participation [41]. In contrast, family obligations, such as caregiving roles, were commonly reported as barriers limiting time for PA [42–44]. This cultural barrier seems to be more prevalent among the Chinese population than in other groups, as traditional Chinese family values prioritise family responsibilities over individual needs [45], highlighting the need for culturally sensitive and flexible PA interventions that accommodate family commitments. Moreover, participants are also motivated to engage in PA to maintain independence and avoid burdening their family members. In traditional Chinese culture, children are expected to care for their parents in times of illness, reflecting filial piety [46]. Consequently, many older adults prioritise their health to reduce the caregiving burden on their children [47]. Additionally, some participants viewed family PA, such as walking and hiking, as a way to strengthen family bonds, as it provides an opportunity for high-quality interaction and communication, thereby promoting family harmony [48].

### Motivation

Consistent with previous research [49], belief in the health benefits of PA emerged as a primary motivator for participation. Many participants recognised PA as essential for maintaining mobility, preventing chronic diseases, and fostering overall well-being. However, our study further suggests that for older Chinese adults, these beliefs are not only grounded in personal health but also in cultural values such as self-sufficiency and reducing the caregiving burden on family members [46].

Despite acknowledging the benefits, many participants expressed concerns about potential dangers, such as falls, joint pain, or overexertion. These fears led to hesitancy in engaging in more demanding PA, particularly among those with existing health conditions. Similar patterns have been observed in other ethnic minority groups, where risk aversion and fear of injury act as deterrents to exercise participation [38]. Such concerns highlight the need for culturally appropriate interventions that build knowledge, self-efficacy, and intrinsic motivation, alongside safe and appropriately tailored exercise programmes for older adults, with education on injury prevention to build confidence and mitigate fear-related barriers.

Many participants described PA as a natural part of daily life—activities like walking, was done effortlessly, highlighting the role of habit in sustaining long-term engagement [50]. Promoting regular, low-barrier activities may support automatic participation and reduce reliance on motivation. Emotions also influenced behaviour. Positive feelings, such as relaxation and enjoyment from dancing encouraged PA, while boredom, frustration, or embarrassment in unfamiliar settings discouraged it. This aligns with findings that positive emotions boost, and negative emotions hinder, PA adherence [51]. Offering culturally relevant and enjoyable options may help maintain long-term motivation.

### Practical implications of the findings

This study has several practical implications. First, this study highlights substantial variability in PA behaviours among older Chinese adults in the UK, indicating that a one-size-fits-all approach to PA promotion is unlikely to be effective. Second, many identified barriers and facilitators, including limited awareness of PA guidelines, health concerns, access to safe environments, and social support, are shared with the wider older adult population. Accordingly, established PA promotion strategies for older adults, such as education, confidence-building, graded activity, and flexible programme design, are likely to be applicable within this community. Third, the findings highlight culturally specific influences, including language preferences, collectivist values, and reliance on familiar social networks, which shaped where and with whom participants felt comfortable being physically active. Community-centred approaches delivered through trusted Chinese associations, alongside culturally resonant activities and flexible digital or hybrid models, may help reduce opportunity-related barriers. Finally, the identified barriers and facilitators align with the COM-B model, supporting use of the Behaviour Change Wheel to develop targeted interventions that combine evidence-based strategies with culturally tailored adaptations for equitable and sustainable PA participation.

### Strengths and limitations

This study is among the first to explore the barriers and facilitators to PA participation among older Chinese adults in the UK, addressing an important research gap. A key strength lies in its exploratory design, which began without a pre-defined theoretical framework but ultimately aligned well with the COM-B model, reinforcing its relevance for understanding PA behaviours in this population. Another strength is the inclusion of a diverse sample, comprising both Cantonese- and Mandarin-speaking participants, as well as individuals with varying levels of PA engagement.

However, this study also has several limitations. Data analysis was primarily conducted by the lead researcher, although regular team discussions were used to reflect on interpretations and enhance analytic credibility. The use of convenience sampling and a small sample may limit generalisability. Despite efforts to recruit diverse participants, the sample included more female and physically active individuals, while more sedentary adults were less likely to participate, introducing potential selection bias. Future studies should use targeted recruitment strategies to better engage less active older adults.

## Conclusion

Older Chinese adults in the UK exhibited varying levels of PA and faced different barriers, highlighting the importance of considering their diverse needs. The factors influencing PA engagement among older Chinese adults in the UK relate to capability, opportunity, and motivation, aligning with the COM-B framework with embedded culturally sensitive factors such as health beliefs, family obligations and traditional collective values. These findings provide evidence to support the design of effective strategies using the Behaviour Change Wheel, by considering these three factors to promote PA within this population.

## Supplementary Information

Supplementary Material 1.

Supplementary Material 2.

Supplementary Material 3.

## Data Availability

Examples in the form of quotations from the generated and analysed data during this study are included in this published article.

## Abbreviations

COM-B framework: Capability, opportunity and motivation-behaviour framework
BCW: Behaviour Change Wheel
PA: Physical activity
PCIE: Public and Community Involvement and Engagement
WHO: World Health Organization
UK: United Kingdom
