# Supplementary material for: Barriers and facilitators to physical activity among older Chinese adults in the UK: a qualitative descriptive study
Source: BMC Geriatr. 2026 Jun 4;26:1026. doi: 10.1186/s12877-026-07660-y (PMC13460021; doi:10.1186/s12877-026-07660-y)
Supplement: Supplementary file 2 [file 12877_2026_7660_MOESM2_ESM.docx]

**Design and Optimisation of a Physical Activity Promotion Intervention for Older Chinese Diasporas in the UK: A Qualitative Study**

Semi-structured interviews will be used to collect data. Participants will be invited to take part in either a one-to-one or group-based interview (e.g., some may prefer to attend together with spouses or friends who are also eligible for the study), based on their preference. Interviews can be conducted either face-to-face or via virtual platforms such as Teams or Zoom, depending on participants’ preference. Individual interviews are expected to last up to 90 minutes. Interviews will be conducted in Chinese (Cantonese or Mandarin) or English, depending on the language the participant feels most comfortable using. All interviews will be conducted by the lead researcher. A research assistant fluent in Cantonese will be present to assist with translation if needed. All interviews will be audio-recorded to ensure accuracy.

Prior to the interview, participants will receive a participant information sheet outlining the study details and will be given sufficient time to read it. These details will be reiterated before the interview begins, and participants will again be asked whether they wish to take part. If they agree, written informed consent will be obtained before proceeding.

At the end of the interview, the researcher will thank participants for their time and ask if they have anything further to add. A gift voucher will then be provided.

**Interview Guide**

**Demographic questions**

I will first ask some demographic questions as recommended by PROGRESS-Plus, such as age, gender, years living in the UK, education, occupation, language, religion, socioeconomic status, marital status, place of residence, and current physical activity level.

I will then follow the pre-prepared interview topic guide to ask questions.

- Topic one: older Chinese diasporas’ experience of attending a physical activity in the UK. I will begin with questions such as *‘What kind of physical activity are you currently doing?’*, and *‘Why have you chosen this kind of physical activity?’,* ‘*What does physical activity/exercise mean to you?’.*
- Topic two: older Chinese diasporas’ perceived barriers and facilitators of attending physical activities in the UK. I will ask participants questions, such as *‘What motivates you to do the kinds of physical activity you currently do?’, ‘What has kept you from attending physical activities?’.*
- Topic three: the features of the ideal physical activity promotion intervention from the views of older Chinese diasporas. Questions will be asked, such as ‘*What will an ideal physical activity promotion intervention look like from your view?’.*
