# Supplementary material for: Barriers and facilitators to physical activity among older Chinese adults in the UK: a qualitative descriptive study
Source: BMC Geriatr. 2026 Jun 4;26:1026. doi: 10.1186/s12877-026-07660-y (PMC13460021; doi:10.1186/s12877-026-07660-y)
Supplement: Supplementary file 3 [file 12877_2026_7660_MOESM3_ESM.docx]

**Appendix**

**Appendix 1. COREQ_Checklist**

**Appendix 2. Demographic Questionnaire**

**Please answer the following questions. All information will be kept confidential.**

1. What is your age (in years)? ____ years

2. Years living in the UK. How many years have you lived in the UK? ___ years

3. What is your gender? ☐ Female; ☐ Male; ☐ Prefer not to say

4. What is your current marital status? ☐ Married; ☐ Divorced; ☐ Widowed; ☐ Single / never married; ☐ Prefer not to say

5. What is your first (main) spoken language? ☐ Mandarin; ☐ Cantonese; ☐ Other Chinese dialect (please specify): __________☐ Other (please specify): __________

6. What is your current employment status? ☐ Retired; ☐ Working part-time; ☐ Working full-time; ☐ Not working (other reasons)

7. In general, how would you rate your health? ☐ Very good; ☐ Good; ☐ Fair; ☐ Poor; ☐ Very poor

8. How often do you perform physical activity? ☐1 < time each week; ☐ 2-3 times each week;

☐ > 4 times each week

**Appendix 3. Initial interview topic guide**

Topic one: Older Chinese adults’ experience of attending a physical activity in the UK.

I will begin with questions such as

- *What kind of physical activity are you currently doing?*
- *Why have you chosen this kind of physical activity?*
- *What does physical activity/exercise mean to you?*

Topic two: older Chinese diasporas’ perceived barriers and facilitators of attending physical activities in the UK.

I will ask participants questions, such as

- *What motivates you to do the kinds of physical activity you currently do?*
- *What are the facilitators for you to engage in physical activity?*
- *What has kept you from attending physical activities?*

Topic three: the features of the ideal physical activity promotion intervention from the views of older Chinese diasporas.

I will ask participants questions, such as

- *What do the ideal physical activity promotion strategies look like from your view?*

**Appendix 4. The thematic approach used for developing Theme 1 and 2 related to Capability**

**Appendix 5. The thematic approach used for developing Theme 3 and 4 related to opportunity**

**Appendix 6. The thematic approach used for developing Theme 5 and 6 related to motivation**
